# Presence and Severity of Lewy Body Pathology is Modulated by Sex and ApoE4 Zygosity in a Neuropathologically‐Confirmed Alzheimer’s Disease Cohort

**DOI:** 10.1002/alz.088854

**Published:** 2025-01-09

**Authors:** Mila Valcic, Marc A Khoury, Luis R Fornazzari, Nathan W Churchill, Tom A. Schweizer, Corinne E. Fischer, David G. Munoz

**Affiliations:** ^1^ Keenan Research Centre for Biomedical Science, Li Ka Shing Knowledge Institute, St. Michael’s Hospital, Toronto, ON Canada; ^2^ Faculty of Medicine, Department of Neurology, University of Toronto, Toronto, ON Canada; ^3^ Temerty Faculty of Medicine, Division of Neurosurgery, University of Toronto, Toronto, ON Canada; ^4^ Division of Neurosurgery, St. Michael’s Hospital, Toronto, ON Canada; ^5^ Institute of Biomaterials and Biomedical Engineering, University of Toronto, Toronto, ON Canada; ^6^ Keenan Research Centre for Biomedical Science, Li Ka Shing Knowledge Institute, St. Michael's Hospital, Toronto, ON Canada; ^7^ Institute of Medical Sciences, University of Toronto, Toronto, ON Canada; ^8^ Faculty of Medicine, Department of Psychiatry, University of Toronto, Toronto, ON Canada; ^9^ Temerty Faculty of Medicine, Department of Laboratory Medicine and Pathobiology, University of Toronto, Toronto, ON Canada; ^10^ Division of Pathology, St. Michael’s Hospital, Toronto, ON Canada

## Abstract

**Background:**

The APOE ε4 allele (ApoE4) is a known genetic risk factor for developing Alzheimer’s disease (AD). Previous work by our group demonstrated that female ApoE4 homozygotes with Lewy body (LB) pathology were significantly more likely to experience psychosis compared to female ApoE4 non‐carriers, whereas there was no dose‐dependent difference found in males (Valcic et al., 2022). The objective of this study was to examine LB pathology presence and severity in males and females across ApoE4 status, with a particular interest in female ApoE4 homozygotes.

**Method:**

Demographics, APOE genotype, and LB pathology data was collected in individuals with neuropathologically‐confirmed AD (n=1,234) from the National Alzheimer’s Coordinating Center (NACC). Bayesian Multilevel Regression was used to predict the probability of LB pathology presence in males and females across ApoE4 status (0, 1, 2 ε4 alleles). An Ordinal Regression was used to examine the relationship between LB pathology severity (brainstem‐predominant (mild), limbic or amygdala‐predominant (moderate), neocortical‐predominant (severe)) and ApoE4 status, stratifying by sex.

**Result:**

While males had a higher probability of exhibiting LB pathology compared to females, after stratifying by ApoE4 status, female ApoE4 homozygotes showed the highest probability of LB pathology at a 91.1% Highest Density Interval (91.1%HDI[0.12: 0.95]) across both sexes. Within females, ApoE4 homozygotes showed a significantly higher probability of having moderate to severe LB pathology relative to other groups (p =.02). Within males, there was no significant relationship between ApoE4 status and LB pathology severity.

**Conclusion:**

This study revealed that sex and zygosity modulate the effect of ApoE4 on presence of LB pathology and LB severity in neuropathologically‐confirmed AD patients. Specifically, female ApoE4 homozygotes had the highest probability of having LB pathology, as well as greater LB pathology severity. Future work should explore the association between LB pathology severity and psychosis while stratifying for sex and ApoE4 status.

**Reference**: Valcic, M., Khoury, M. A., Kim, J., Fornazzari, L., Churchill, N. W., Ismail, Z., De Luca, V., Tsuang, D., Schweizer, T.A., Munoz, D.G, & Fischer, C. E. (2022). Determining Whether Sex and Zygosity Modulates the Association between APOE4 and Psychosis in a Neuropathologically‐Confirmed Alzheimer’s Disease Cohort. Brain Sciences, 12(9), 1266.

